# Pose estimation of differential drive robots using deep learning and raw sensor inputs

**DOI:** 10.1038/s41598-025-25207-w

**Published:** 2025-11-21

**Authors:** Gullu Boztas, Mustafa Can Bingol, Omur Aydogmus, Musa Yilmaz

**Affiliations:** 1 Faculty of Technology, Department of Electrical and Electronics Engineering, Firat University, Elazig, 23200 Turkey; 2 Faculty of Engineering-Architecture, Department of Electrical and Electronics Engineering, Burdur Mehmet Akif Ersoy University, Burdur, 15100 Turkey; 3 Faculty of Technology, Department of Mechatronics Engineering, Firat University, Elazig, 23200 Turkey; 4 Bourns College of Engineering, Center for Environmental Research and Technology, University of California at Riverside, Riverside, CA 92521 USA; 5 Department of Electrical and Electronics Engineering, Batman University, Batman, 72100 Türkiye

**Keywords:** IMU Sensor, Position estimation, Raw sensor data, Mobile robot, Engineering, Mathematics and computing

## Abstract

This paper presents an estimation method for determining the position and orientation of a real mobile robot using raw data from an Inertial Measurement Unit (IMU) sensor, alongside linear and angular velocities obtained from simulation. The dataset was collected using a real TurtleBot3 differential drive wheeled mobile robot in the ROS-Gazebo simulation environment, encompassing 2018 routes-2009 from simulation and 9 from real-world experiments-each consisting of five randomly generated waypoints. To improve the accuracy of the estimation models, noise from the real IMU sensor was incorporated into the input data, and velocities derived from the pure pursuit algorithm were also included. Convolutional Neural Network (CNN), Long Short-Term Memory (LSTM), Gradient Boosting (GB), and Random Forest (RF) models were employed to estimate the robot’s position and orientation, and their performance was compared across both simulated and experimental scenarios. The results indicate that the CNN architecture consistently outperforms other models across all routes. Unlike many existing studies, this work directly utilizes raw sensor data without applying any feature extraction techniques, highlighting its novelty and contribution to the field.

## Introduction

Mobile robots have gained widespread popularity in recent decades, witnessing an increasing array of applications. They have transitioned from being merely subjects of research to commercially available products, underscoring the growing significance of studies in this field^1^. The diverse application areas of mobile robots include warehousing^2,3^, package delivery^4^, medical facilities^5^, customer service^6^, emergency response^7^, disaster recovery^8^), space exploration^9^, defense, and entertainment^10^. Mobile robots can be broadly categorized into legged robots and wheeled robots, serving as indoor robots, terrain robots, aerial robots, and underwater robots. Wheeled robots, especially those with easy control and high position accuracy, dominate the application landscape. Within wheeled mobile robots, there are distinctions between non-holonomic and holonomic types. Mathematical representation involves a set of non-integratable first-order differential constraints, assuming no slipping during movement. Developing a suitable controller for stabilization and trajectory tracking of non-holonomic robots is often a challenging task^11^. Accurate localization is a critical requirement for mobile robots^12^. While high-cost products like vision-based and lidar-based localization systems are commonly used for achieving precision, internal measurements offer a cost-effective alternative, enhancing accuracy and reliability. The Inertial Measurement Unit (IMU) sensor emerges as a favorable choice for predicting the robot’s position or localization due to its relatively low cost and easy installation on mobile robots. Despite its advantages, IMU sensors cannot provide absolute position or localization information. IMU sensors typically include gyroscope, accelerometer, and magnetometer as optional components, enabling the measurement of orientation, angular velocity, and linear acceleration in 3D. Consequently, the position or localization information must be calculated using the sensor’s data. Various approaches have been proposed in the literature for position and location estimation using IMU sensors, including combining IMU with LiDAR and encoder measurements to estimate robot position on complex terrains^13^.

Moreover, artificial neural networks are employed to extract valuable information from sensor data across various domains. In a study by Bangaru et al.^14^, data from electromyography and IMU sensors on a wristband developed for workers were classified using an artificial neural network, facilitating the analysis of worker movements. Convolutional Neural Network (CNN), an architecture comprising specialized layers, has found applications in diverse areas. Ding et al.^15^ utilized a CNN to analyze data from an accelerometer sensor placed in cargo boxes, determining the cause of box damage. Similarly, Suri and Gupta^16^ employed a CNN architecture to classify data from a wearable IMU, achieving sign language recognition. Long Short-Term Memory (LSTM), another artificial neural network architecture with specialized layers akin to a recurrent neural network (RNN), has been used in Peng et al.’s^17^ study to classify the behaviors of Japanese black cattle based on signals from video and IMU data. Studies combining these architectures are prevalent in the literature. For instance, Kim et al.^18^ investigated position estimation from IMU data using both CNN and LSTM structures. In another study, Zhao and Obonyo^19^ achieved posture estimation by utilizing data from multiple IMU sensors on a worker with both CNN and LSTM structures. Various machine learning algorithms are employed in processing sensor data. Qaroush et al.^20^ classified data received from IMU sensors on the hand using machine learning methods to recognize Arabic sign language. The Random Forest (RF) algorithm, a popular choice, was used by Kareem et al.^21^ to recognize walking behaviors of mobile phone users through gyroscope data. Additionally, Langroodi et al.^22^ performed action recognition using an RF-based classifier with data from IMU and GPS sensors on construction machines. The Gradient Boosting (GB) algorithm, frequently utilized in the literature, was employed by Tan et al.^23^ to predict ground reaction force using IMU and GB algorithms. Liu et al.^24^ measured runner performances with IMU and artificial intelligence algorithms, including CNN, GB, and MLP. Studies combining these two machine learning algorithms are also present, as seen in the work of Kiangala and Wang^25^. In their study, a regression model using the GB algorithm was followed by the creation of a classification model using the RF algorithm and the output of the regression model, exemplifying the integration of both algorithms in data classification after a regression process using the GB algorithm. In this study, the objective was to estimate the position and orientation of a differential drive wheeled mobile robot (DDWMR) using raw data from an IMU, in conjunction with reference linear velocity and reference angular velocity. The training and validation datasets were collected within the Gazebo robot simulator environment, incorporating IMU sensor noises from the real robot to enhance simulation realism. To reduce the sim-to-real gap, real IMU noise characteristics were added to the simulation data. The noise was modeled as zero-mean Gaussian noise based on empirical measurements from the physical IMU. The standard deviation was estimated from stationary sensor recordings and applied independently to each axis. This ensured that the simulated IMU outputs reflected both the sensor bias and high-frequency variability observed in the real device. The magnitude of the added noise corresponded to 0.02 $m/s^2$ for accelerometer channels and 0.001 *rad*/*s* for gyroscope channels, consistent with the noise levels reported in the sensor datasheet. The dataset comprises 2009 different routes, each containing 12 inputs and 3 targets, with 5 randomly generated waypoints per route. The input data include *x*, *y*, *z*, *w* quaternion orientation, angular velocities of *x*, *y*, *z*, linear acceleration of *x*, *y*, *z*, and reference linear and angular velocities. The target outputs consist of the robot’s position *x*, position *y*, and orientation $\theta$, measured using an odometry sensor. CNN, LSTM, GB, and RF models were trained and validated using this dataset. To assess model performance, a test dataset comprising 9 routes was collected from a real TurtleBot3 DDWMR. The analysis of model performance on the test dataset revealed consistent results between simulation and experimentation for all tested routes, with the CNN model exhibiting the best overall accuracy based on performance metrics.

## Materials and methods

### Problem descriptions

In this study, the TurtleBot3-Burger was used to collect data for the training and testing of estimation algorithms. Widely recognized in the literature, this robot is favored for its open-source features catering to developers. The TurtleBot3-Burger is a non-holonomic mobile robot characterized by constraint velocities^26^. Its technical specifications include a maximum translational velocity of 0.22 m/s, a maximum rotational velocity of 162.72 $^\circ /s$, and a maximum payload capacity of approximately 15 kg. The robot’s dimensions are 138$\times$178$\times$192 mm, with a weight of 995 g, including a single board computer, battery, and default sensors. It can traverse a maximum step of 10 mm. The mechanical and dynamic properties of the TurtleBot3-Burger can be effectively simulated using the Gazebo simulator-an open-source 3D simulation environment. This simulator provides a realistic depiction of robot dynamics and rendering views in complex indoor and outdoor environments. Supporting various physics engines such as ODE, Bullet, Simbody, DART, etc., the Gazebo simulator allows for the simulation of both the developed robots and algorithms, providing a realistic scenario for testing and development. Moreover, the simulator offers real-time sensor data with noise, and all actuators and sensors can be connected via TCP/IP as a remote interface service^1^. In this study, the TurtleBot3-Burger was controlled using a traditional pure pursuit algorithm (PPA). The control algorithm was implemented in MATLAB and communicated with the robot operating system (ROS). ROS distribution Noetic served as a bridge between the MATLAB control algorithm and the real robot/Gazebo simulator environments.

**Fig. 1:**
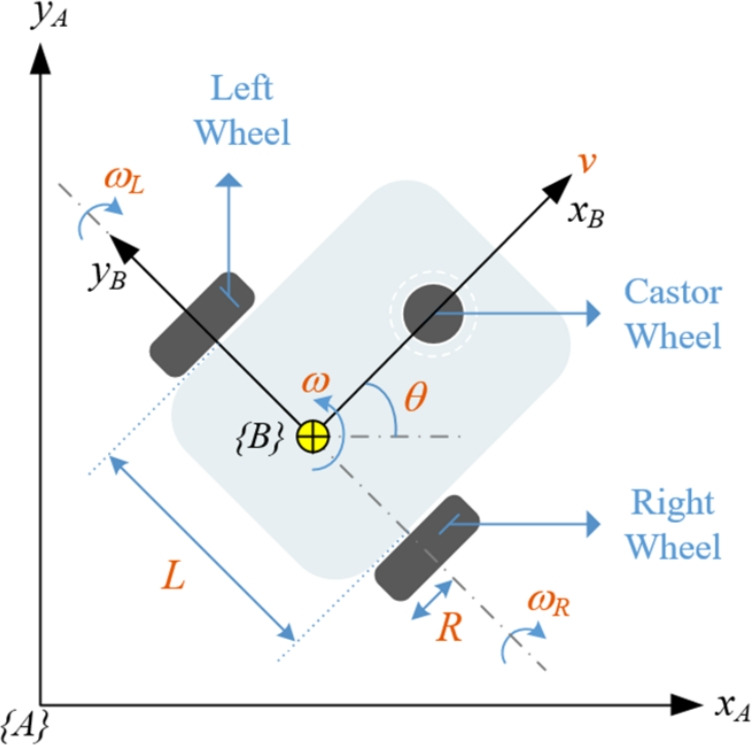
Free body diagram of differential drive wheeled mobile robot.

The kinematics equations of the DDWMR can be derived by using the parameters given in Fig. 1 The robot motion is represented by linear velocity *v* (m/s) and angular velocity *w* (rad/s). The equations of forward kinematics are given in Eqs. (1) and (2). The inverse kinematics of the robot is given in Eq. (3) and (4).

**Fig. 2:**
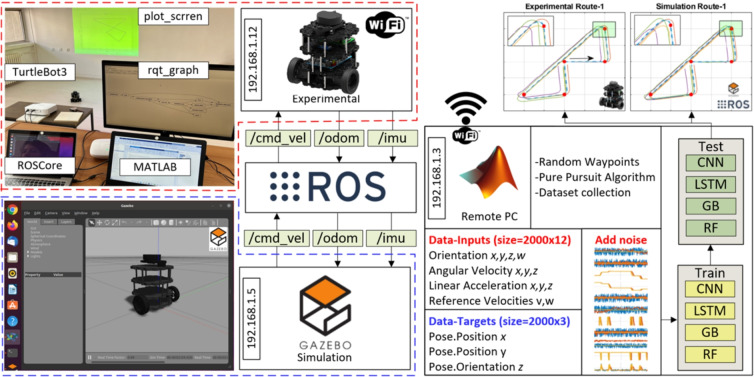
Experimental and simulation framework for development and testing of algorithms using TurtleBot3.

1 $$\begin{aligned} & \begin{aligned} v = \frac{R}{2}\left( \omega _R + \omega _L\right) \end{aligned} \end{aligned}$$

2 $$\begin{aligned} & \begin{aligned} \omega = \frac{R}{L}\left( \omega _R - \omega _L\right) \end{aligned} \end{aligned}$$

3 $$\begin{aligned} & \begin{aligned} \omega _L = \frac{1}{R}\left( v - \frac{\omega L}{2}\right) \end{aligned} \end{aligned}$$

4 $$\begin{aligned} & \begin{aligned} \omega _R = \frac{1}{R}\left( v + \frac{\omega L}{2}\right) \end{aligned} \end{aligned}$$

where *R* is the wheel radius of the motorized wheels, *L* is the wheelbase of the robot, *wL* and *wR* are the angular velocity of left and right wheel, respectively. The PPA requires the actual positions of the robot *x*, *y*, *z* and the waypoints *xy* in order to determine the references of the linear and actual velocities. The kinematic model of the robot can be expressed as shown in Eq. 5.

5 $$\begin{aligned} \begin{aligned} \begin{bmatrix} \dot{x} \\ \dot{y} \\ \dot{\theta } \end{bmatrix} = \begin{bmatrix} cos(\theta ) & -sin(\theta ) & 0 \\ sin(\theta ) & cos(\theta ) & 0 \\ 0 & 0 & 1 \end{bmatrix} \begin{bmatrix} v \\ 0 \\ \omega \end{bmatrix} \end{aligned} \end{aligned}$$

The PPA calculates the distance between robot pose and the waypoints. In addition, it calculates the course of the path. The equations of the PPA are given in Eq.6 and Eq.7.

6 $$\begin{aligned} & \begin{aligned} d = \sqrt{\left( x_{ps}-x_{wp}\right) ^2+\left( y_{ps}-y_{wp}\right) ^2} \end{aligned} \end{aligned}$$

7 $$\begin{aligned} & \begin{aligned} c = atan2\left( \left( y_{wp}-y_{ps}\right) ,\left( x_{wp}-x_{ps}\right) \right) \end{aligned} \end{aligned}$$

where *d* is the distance between robot pose and the reference waypoint. *c* is the course of path. $x_{ps}$ any $y_{ps}$ are the robot positions on the *xy*-plane. $x_{wp}$ any $y_{wp}$ are the waypoints on the *xy*-plane.

**Fig. 3:**
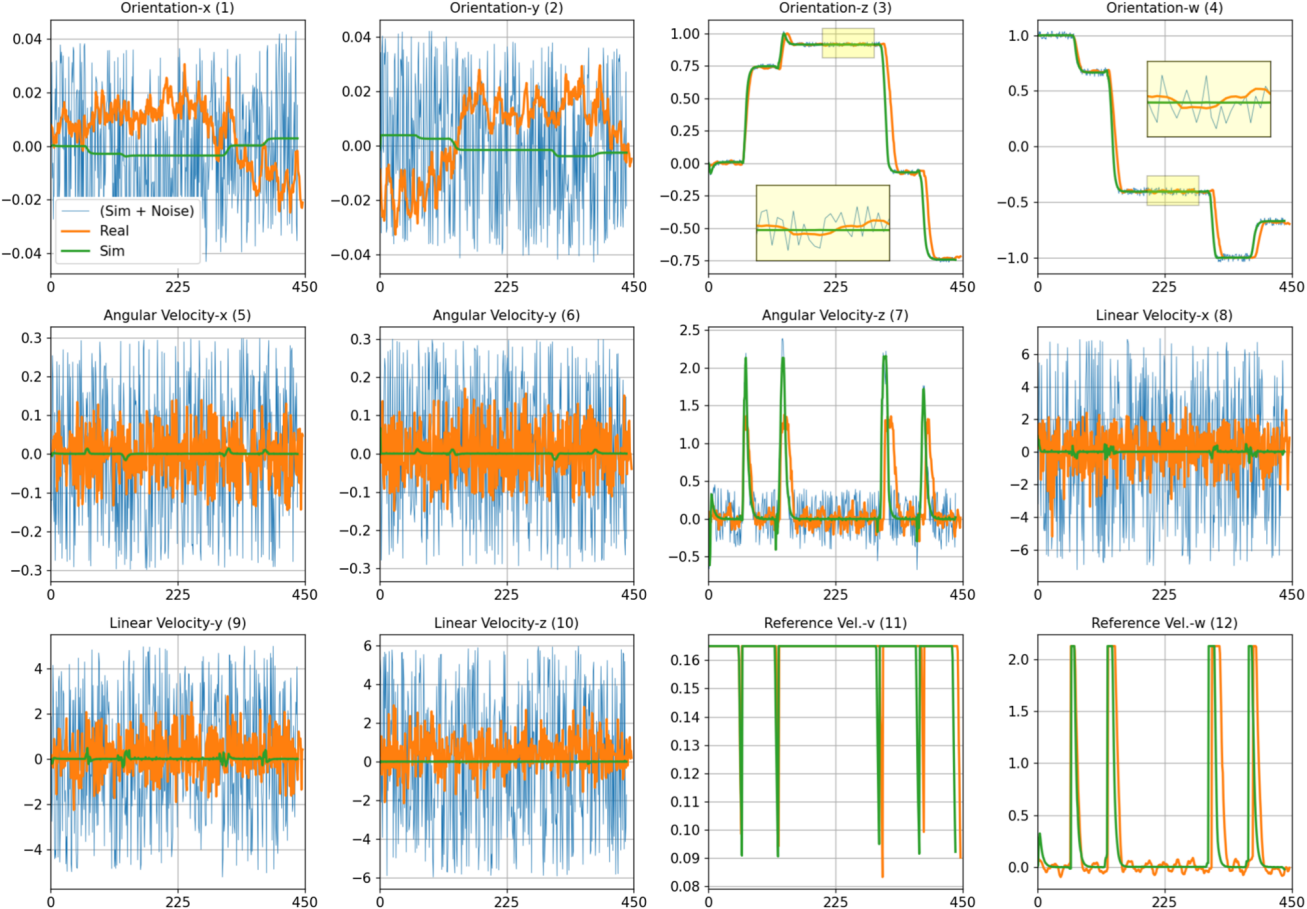
Comparison of IMU sensor outputs from simulation with added noise (Sim + Noise), real experimental data (Real), and ideal simulation data (Sim) during the training process. Plots include orientation (quaternion) components, angular velocities, linear velocities, and reference velocities across x, y, z, and w axes. Highlighted areas indicate regions of higher noise and discrepancies between real and simulated data.

The block diagram of the system used for data collection is shown in Fig. 2. The orientation *x*, *y*, *z*, *w*, angular velocity *x*, *y*, *z*, linear acceleration *x*, *y*, *z*, and references of linear and angular velocities are used as inputs for training models. Each input is reproduced by using one-unit delay and two unit delays. Thus, the input size was increased from 12 to 36. The robot was traveled for 5 randomly assigned waypoints using PPA. This situation was repeated 2009 times to create a ($t\times$2009$\times$12$\times$3) dataset. The real IMU sensor noise were added for all simulation inputs in order to obtain realistic results for training procedure. The output signals of the real and simulation IMU sensors are given in Fig. 3.

### Algorithms for prediction

The collected dataset was divided into three different datasets for training, validation and testing. All sensor inputs and target values were normalized prior to training. Each input channel was standardized using the mean and standard deviation computed from the training set, and the same transformation was applied to the validation and test sets. The same procedure was used for the target values. This normalization ensured that all features were on a comparable scale, which improved training stability and convergence. The dataset used in the CNN algorithm have 3$\times$12$\times$N inputs and 3$\times$N outputs. Multiplying by 3 at the input represents the results at moments *t*, $t-1$, and $t-2$. The IMU provides the data of quaternion orientations (*x*, *y*, *z*, *w*), angular velocities (*x*, *y*, *z*), and linear acceleration (*x*, *y*, *z*). In addition, the data of the reference linear and angular velocities are determined by the PPA. Thus, 10 different data from IMU sensor and 2 data from PPA were used as inputs for the proposed estimation algorithm. N is the input and output component of the dataset represents the number of the total steps in the dataset. Additionally, the number 3 in the output of the dataset presents the position variable on the x-axis $\Delta (x) = x(t) - {x(t - 1)}$ and y-axis $\Delta (y) = y(t) - {y(t - 1)}$, and the orientation angle $\theta$ variables of the mobile robot. The outputs of the dataset used in GB, LSTM and RF algorithms were the same and the input size was 1$\times$12$\times$N. The number 1 in the input datasets represents the results at time *t*. GB, LSTM, and RF algorithms are also trained using data having inputs in 3$\times$12$\times$N size just like CNN. However, significant results were not obtained in this situation. Dataset having input in the 1$\times$12$\times$N size was used in other algorithms except for CNN due to the computational time of the CPU. In addition, datasets with an input size of 3$\times$12$\times$N were used in the CNN algorithm to be more reliable of the convolution process. The CNN architecture is shown in Algorithm 1. For the CNN, all convolutional layers used kernel size 3$\times$3, stride 1, and “same” padding, followed by ReLU activations. The final dense layers had 128 ReLU units and 3 linear units. For the LSTM model, we used a single LSTM layer with 1024 hidden units (tanh activation, sigmoid recurrent activation), followed by a dense layer of 1024 ReLU units and a linear output layer. Both models were trained with the Adam optimizer (learning rate 1e-4) and MSE loss. Dropout and Gaussian noise layers were tested but not used in the final version. The filter sizes (32, 64) and LSTM hidden dimension (1024) were selected after preliminary experiments balancing accuracy and computational complexity. Smaller models showed underfitting, while larger ones increased computation without noticeable accuracy gains.

The data is reshaped as 1$\times$N size in the Flatten layer after performing the necessary feature extraction in the Convolution Layer^27^. The reshaped data is subjected to mathematical operations in Dense layers^28^ to produce output. The dense layer was used to establish associations between all inputs and outputs, where each neuron connects to every neuron in the preceding layer. This layer serves to connect the outputs of specialized Convolutional and LSTM layers. The mathematical relationships within this layer, along with learning algorithms, are utilized to minimize residual errors. The LSTM structure being another deep learning structure is given in Algorithm 2. LSTM layer is a specialized architecture which records or forgets the input data depending on their relevance to one another and the result^29^. Detailed information about the layers and activation functions in Algorithm 1 and Algorithm 2 can be found in^30,31^. Adam optimization algorithm was used when both CNN and LSTM architectures were trained. In this study, ADAM, a derivative-based learning algorithm, was used. The ReLU activation function was employed to expedite the calculation of derivative operations. The total epochs of the training took 25. The learning rate applied throughout the training was dynamically changed by using learning rate decay function algorithm. Each neural network was trained for 25 epochs with a batch size of 2048. A learning rate scheduler was applied, using 1$\times$10e-3 for the first 10 epochs, 1$\times$10e-4 for epochs 10-19, and 1$\times$10e-5 for epochs 20-25. Early stopping was not employed, since preliminary experiments indicated that 25 epochs with scheduled decay provided stable convergence without overfitting. In this study, the models were trained using differences in positions. These position variances were observed to be quite small in some cases. Consequently, the learning rate was adjusted by decreasing it during training to move closer to the global minimum. ReLU activation is employed in the CNN layers because it is widely adopted in convolutional architectures, providing robustness against vanishing gradients and enabling efficient training. In the LSTM, the tanh activation is used for the hidden state since it normalizes outputs to the range [−1,1], which contributes to stable temporal learning dynamics. The recurrent gates utilize the standard sigmoid activation to regulate information flow.

**Algorithm 1:**
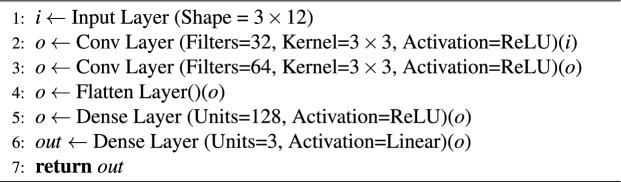
CNN Architecture

**Algorithm 2:**
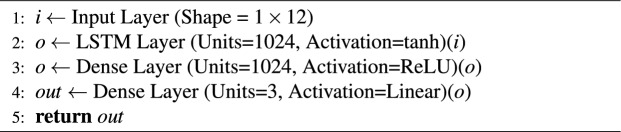
LSTM Architecture

**Algorithm 3:**
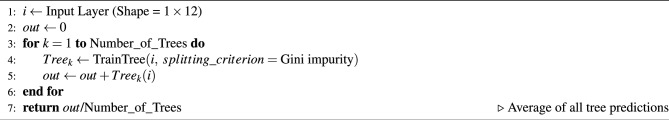
Random Forest (RF)

**Algorithm 4:**
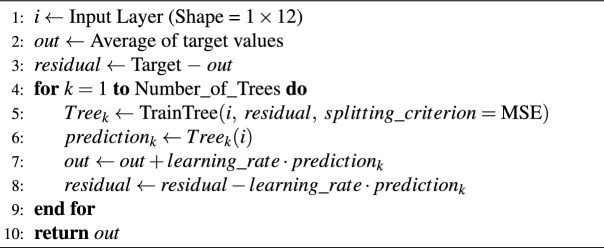
Gradient Boosting (GB)

The RF algorithm is based on the generation of the output by using multiple decision trees in parallel. In regression problems such as this study, RF generates the output value by averaging the outputs of the used trees. Detailed information about the algorithm can be found in^32^. In this study, the total number of trees was determined as 25. In addition, there is no limit on the number of leaf nodes. The RF structure was given in Algorithm 3.

The GB algorithm is based on the strong model formed by more than one consecutive weak model. The principle of the algorithm is to create a new estimator by using the residual error from the previous estimator. The detailed information about the algorithm can be found in^32^. In this study, 25 decision trees were used for each output. The each tree was set to have maximum of 10 nodes and the learning rate was chosen as 0.1. Tree numbers of the both RF algorithm and GB algorithm were tested with 1, 10, 25, 50, and 100, respectively. The best result was obtained with 25 as a result of the performance analysis. The GB structure was given in Algorithm 4. For Random Forest and Gradient Boosting, the number of trees was varied in (1, 10, 25, 50, 100). Validation performance improved significantly when increasing from 1 to 25 trees, but showed only marginal gains beyond 25 while increasing computation time. Therefore, 25 trees were selected as a trade-off between accuracy and efficiency.

To investigate the impact of temporal input structure on model performance, an ablation study was conducted by comparing a 1-step CNN model, which uses only the current time step, with a 3-step CNN model that incorporates temporal information from the current and two previous time steps (t, t-1, t-2). The 1-step CNN achieved a training loss of 0.0728 and a test loss of 0.0739, whereas the 3-step CNN significantly outperformed it, with training and test losses of 0.0290 and 0.0282, respectively. These results demonstrate that incorporating a short temporal window substantially improves estimation accuracy by enabling the model to capture short-term temporal dependencies. While the 1-step CNN is computationally lighter, this comes at the cost of reduced predictive performance. This analysis confirms that the superior performance of the CNN model is primarily due to the inclusion of temporal context in the input representation.

## Results

The dataset was generated by recording the robot’s traversal along 2,018 distinct routes. In this study, 2,000 routes collected in the simulation environment were used exclusively for training. For validation, the same nine routes that were later executed on the real robot were also evaluated in the simulation environment to ensure consistency between simulated and real-world scenarios. For testing, real-world data collected from the robot operating along these nine routes were directly utilized to assess the model’s performance in real conditions. Approximately 9.1$\times 10^5$ steps were involved in the training routes, while the verification and testing routes comprised approximately 4$\times$ $10^3$ steps. The dataset occupied 97.5 MB of disk space. The training dataset played a crucial role in training the artificial intelligence model. The validation dataset was employed to refine the hyperparameters of the model during the training process. The performance of the trained system was evaluated using the test dataset. Table 1 presents the Mean Absolute Error (MAE) values of the proposed algorithms. The CNN algorithm achieved the best results for both the validation and test datasets. The RF algorithm demonstrated the best performance in the training dataset. Algorithmic performance was statistically compared using only the test dataset. To assess real-time feasibility, the inference performance of CNN, LSTM, GB, and RF models was measured using a synthetic test set. Average inference times and memory usage are summarized in Table 1. CNN achieved 0.93 ms per prediction with 71 MB memory, while LSTM required 3.97 ms with the same memory footprint. Tree-based methods (GB and RF) were orders of magnitude faster (0.001-0.007 ms) and used negligible memory. These results indicate that while deep learning models may offer higher accuracy, tree-based models provide near-instantaneous inference suitable for computationally constrained real-time robotic applications.

**Table 1:** Training, validation, and testing performance of proposed algorithms (MAE in meters).

**Algorithm**	**Training**	**Validation**	**Testing**	**Time / ms**	**Memory / MB**
CNN	0.0288	**0.0281**	**0.0589**	0.930	71.24
GB	0.1045	0.1161	0.3284	0.001	0.00
LSTM	0.0754	0.0814	0.0673	3.974	71.24
RF	**0.0283**	0.0792	0.2005	0.007	0.00

**Fig. 4:**
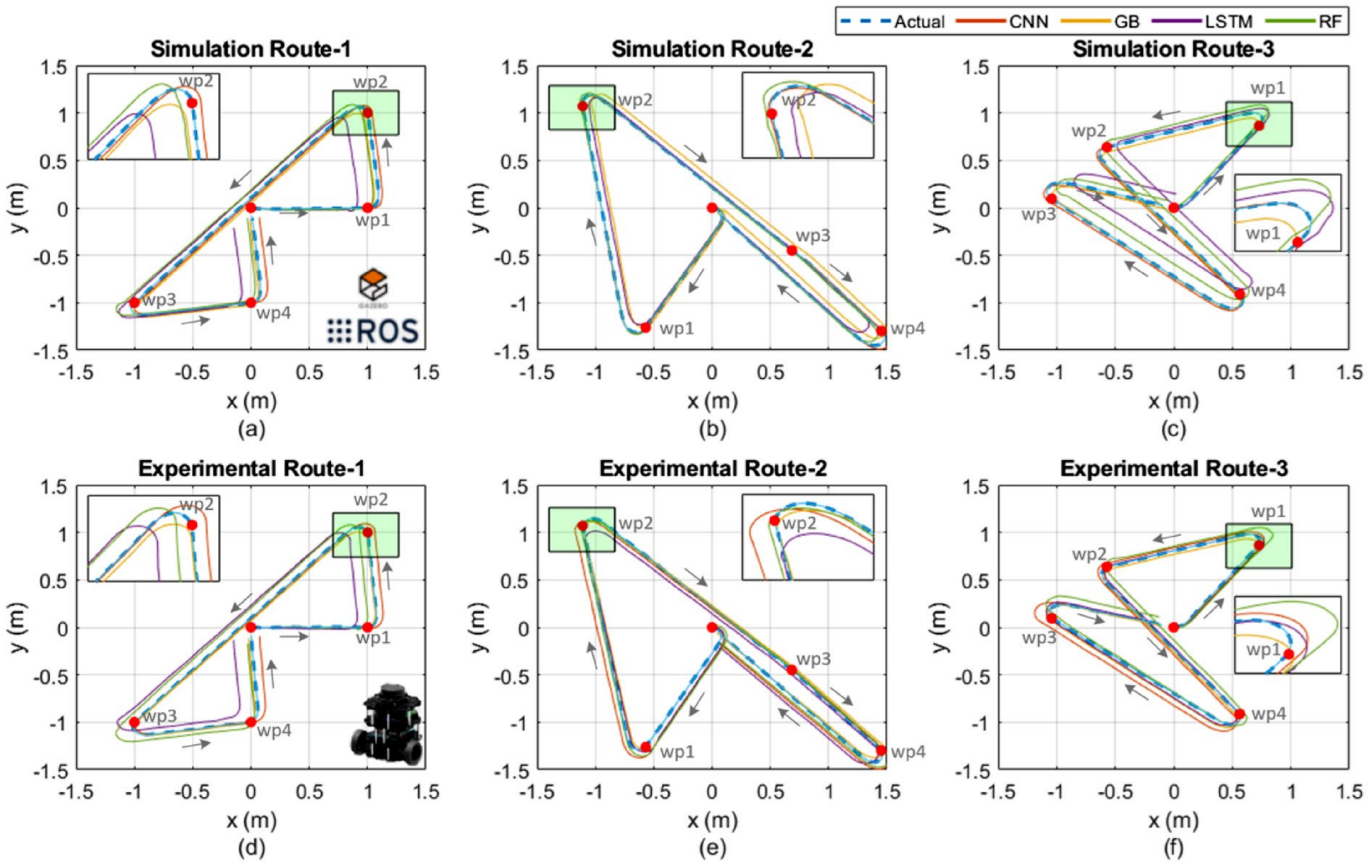
Experimental and simulation results for actual and estimated routes; Route-1 waypoints: [1 0; 1 1; -1 -1; 0 -1; 0 0], Route-2 waypoints: [-0.5692 -1.2615; -1.1086 1.0704; 0.6845 -0.4487; 1.4507 -1.2967; 0 0], and Route-3 waypoints: [0.7281 0.8641; -0.5719 0.6391; 0.5653 -0.9122; -1.0430 0.0951; 0 0] – (**a**) Sim. Route 1, (**b**) Sim. Route 2, (**c**) Sim. Route 3, (**d**) Exp. Route 1, (**e**) Exp. Route 2, (**f**) Exp. Route 3.

Three different routes were selected randomly from the test dataset and the actual and estimated routes traveled by the real and simulation robot are shown in Fig.4. The robot traveled towards CCW (counter clockwise) direction in Fig. 4a and Fig. 4b. The robot traveled CW (clockwise) and CCW directions in the last route as shown in Fig. 4c. The results showed that the estimated routes were best obtained by CNN for simulation and experimental tests.

**Fig. 5:**
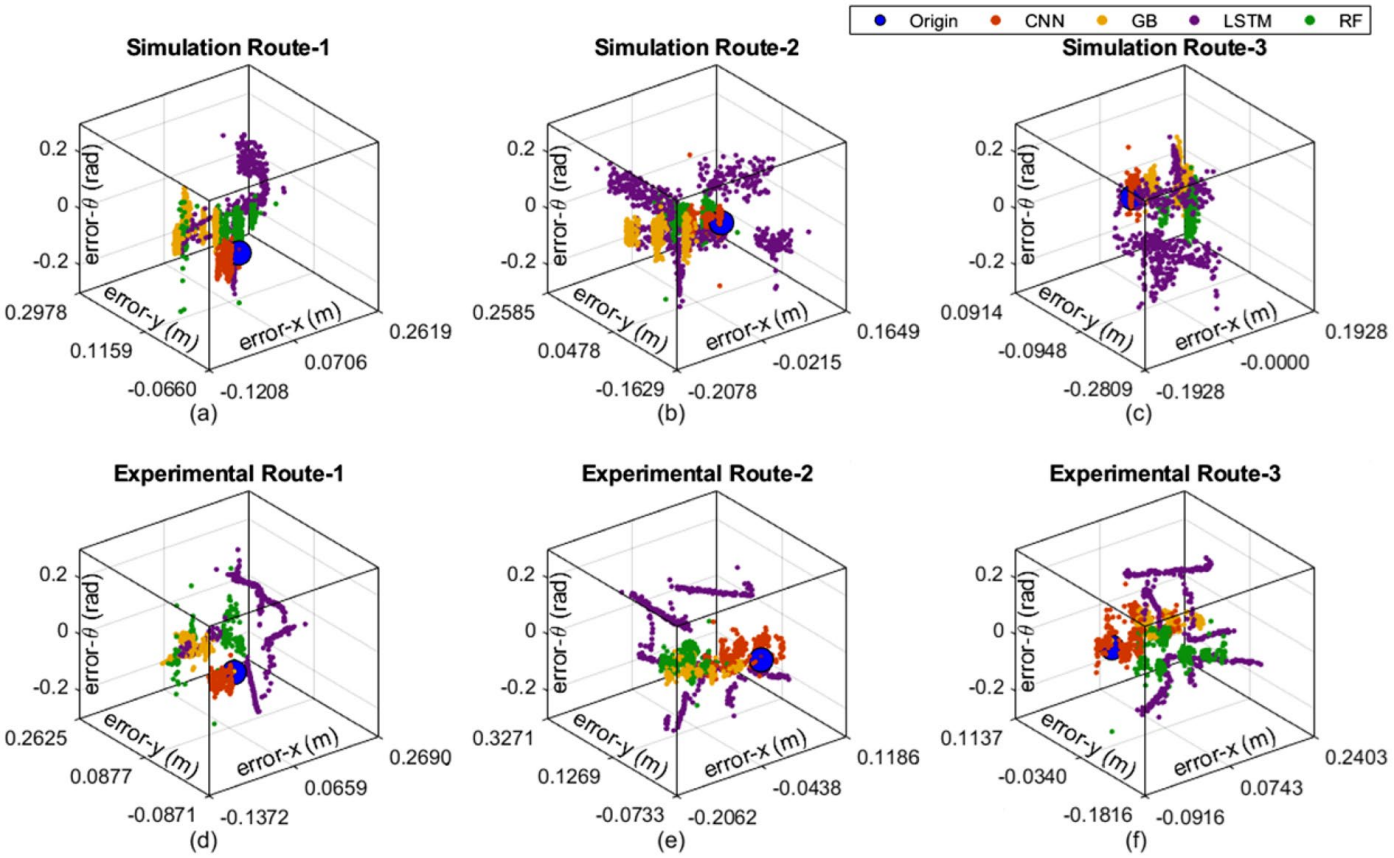
Estimation errors of experimental and simulation results; (**a**) Sim. Route 1, (**b**) Sim. Route 2, (**c**) Sim. Route 3, (**d**) Exp. Route 1, (**e**) Exp. Route 2, (**f**) Exp. Route 3.

The estimation errors of the pose-*x*, pose-*y*, and orientation-$\theta$ are given in Fig. 5 for all models. The blue point was placed at the origin of the coordinates in the figures in order to show easy comparison of the estimation errors. It can be observed that the CNN model gives the values closest to zero errors for all estimation axes. LSTM estimated the far points from the zero error point compared to other estimation models for both simulation and experimental routes.

The performance metrics of the models were calculated by using the average of the error square (MSE), the square root of the average of the error square (RMSE), the mean absolute error of *N* predicting series (MAE), and the coefficient of how well the values fit compared to the actual values ($R^2$).

Table 2 presents a comprehensive performance evaluation of four machine learning models-CNN, GB, LSTM, and RF-across three distinct routes. The models’ predictive accuracy for trajectory coordinates (*x*, *y*) and orientation ($\theta$) was assessed using both simulated (Sim.) and experimental (Exp.) datasets, with performance measured using MSE, RMSE, MAE, and the coefficient of determination ($R^2$). A key and unambiguous finding is the superior performance of the CNN model across all routes, conditions, and evaluation metrics. For simulated data, CNN consistently achieved $R^2$ values exceeding 0.99 and extremely low error rates (e.g., MSE on the order of $10^{-6}$), indicating a near-perfect fit. Although its performance slightly declined when applied to the noisier experimental data, it remained robust, with $R^2$ values consistently above 0.92. In contrast, the GB, LSTM, and RF models constituted a clear second tier, displaying significantly higher error metrics and lower $R^2$ values, generally ranging from 0.60 to 0.84. Another noteworthy observation is that all models performed better on simulated data than on experimental data, a predictable outcome reflecting the idealized conditions of simulations. Notably, the prediction of orientation ($\theta$) was exceptionally accurate for the CNN model, often yielding $R^2$ values above 0.999, suggesting that its architecture is particularly well-suited for capturing rotational dynamics in this context.

**Table 2:** Combined comparison of model performance across three routes (1, 2, and 3), various models, and prediction types (x, y, $\theta$).

Route	Study	Metric	CNN			GB			LSTM			RF		
			x	y	$\theta$	x	y	$\theta$	x	y	$\theta$	x	y	$\theta$
1	Sim.	MSE	1.51E-06	1.37E-06	0.000563	4.74E-05	4.05E-05	0.0208	4.74E-05	4.71E-05	0.0097	4.97E-05	4.68E-05	0.0116
		RMSE	0.0012	0.0012	0.0237	0.0069	0.0064	0.1443	0.0069	0.0069	0.0984	0.007	0.0068	0.1077
		MAE	0.000487	0.000496	0.019	0.002	0.0018	0.1106	0.0013	0.0012	0.0335	0.0016	0.0016	0.0375
		$R^2$	0.9919	0.9909	0.9998	0.6321	0.6714	0.9909	0.6374	0.6037	0.9964	0.7027	0.6756	0.9957
	Exp.	MSE	1.29E-05	8.09E-06	0.000224	4.21E-05	3.91E-05	0.0177	4.13E-05	3.96E-05	0.0049	6.20E-05	4.86E-05	0.000824
		RMSE	0.0036	0.0028	0.015	0.0065	0.0063	0.1332	0.0064	0.0063	0.0703	0.0079	0.007	0.0287
		MAE	0.0023	0.0019	0.0102	0.0028	0.0023	0.1102	0.0027	0.0023	0.01	0.0029	0.0025	0.013
		$R^2$	0.9293	0.9438	0.9999	0.6809	0.658	0.9922	0.6822	0.6425	0.9981	0.6548	0.6752	0.9997
2	Sim.	MSE	6.68E-07	1.18E-06	0.00063	1.99E-05	3.60E-05	0.0406	2.16E-05	4.09E-05	0.0276	1.90E-05	3.66E-05	0.0618
		RMSE	0.000817	0.0011	0.0251	0.0045	0.006	0.2015	0.0046	0.0064	0.1662	0.0044	0.0061	0.2486
		MAE	0.000399	0.000446	0.0174	0.0013	0.0019	0.1172	0.000947	0.0012	0.0359	0.000984	0.0013	0.0399
		$R^2$	0.994	0.9944	0.9998	0.7664	0.7972	0.9827	0.762	0.7633	0.9898	0.8018	0.816	0.9772
	Exp.	MSE	6.81E-06	1.17E-05	0.0658	2.16E-05	3.62E-05	0.0364	2.13E-05	3.36E-05	0.0312	2.33E-05	3.59E-05	0.0402
		RMSE	0.0026	0.0034	0.2565	0.0047	0.006	0.1909	0.0046	0.0058	0.1767	0.0048	0.006	0.2005
		MAE	0.0019	0.0026	0.0278	0.002	0.0029	0.1179	0.0021	0.0029	0.0251	0.0021	0.0029	0.0239
		$R^2$	0.9372	0.9418	0.9762	0.7545	0.7838	0.9845	0.7569	0.7911	0.9885	0.7562	0.8116	0.9853
3	Sim.	MSE	9.82E-07	6.89E-07	0.000659	2.85E-05	1.98E-05	0.0689	4.81E-05	2.91E-05	0.0179	3.00E-05	2.12E-05	0.0446
		RMSE	0.000991	0.00083	0.0257	0.0053	0.0045	0.2625	0.0069	0.0054	0.1338	0.0055	0.0046	0.2111
		MAE	0.000431	0.00042	0.0193	0.0017	0.0014	0.1322	0.0013	0.0011	0.0379	0.0012	0.0011	0.0426
		$R^2$	0.995	0.9948	0.9998	0.8199	0.8293	0.9775	0.6857	0.7156	0.995	0.834	0.8372	0.9874
	Exp.	MSE	1.42E-05	8.70E-06	0.0794	4.75E-05	2.38E-05	0.0408	4.41E-05	1.72E-05	0.0286	5.47E-05	3.21E-05	0.0446
		RMSE	0.0038	0.0029	0.2819	0.0069	0.0049	0.202	0.0066	0.0041	0.169	0.0074	0.0057	0.2113
		MAE	0.0026	0.002	0.0327	0.003	0.0023	0.1296	0.003	0.0022	0.0293	0.0031	0.0024	0.0285
		$R^2$	0.9238	0.9327	0.9788	0.7018	0.7985	0.9873	0.6861	0.8215	0.9923	0.7043	0.7693	0.988

The studies include both simulated (Sim.) and experimental (Exp.) data. Lower is better for MSE, RMSE, MAE; higher is better for $R^2$.

Table 3 presents the mean ± standard deviation of the MSE for all models across the three routes, considering both simulated (Sim.) and experimental (Exp.) data. Each model’s results are summarized in two rows, with the first row showing simulated data and the second row showing experimental data. This format allows for a clear comparison of the variability and accuracy of each model while highlighting the consistency of performance between simulation and real-world conditions. Only MSE is reported in this revised table to focus on a single, widely recognized performance metric.

**Table 3:** Mean ± standard deviation of MSE across three routes for Simulated and Experimental data.

Model	Data	x $(mean \pm std. dev.)$	y $(mean \pm std. dev.)$	$\theta$ $(mean \pm std. dev.)$
CNN	Sim.	$1.05 \times 10^{-6} \pm 3.47 \times 10^{-7}$	$1.08 \times 10^{-6} \pm 2.87 \times 10^{-7}$	$6.17 \times 10^{-4} \pm 4.02 \times 10^{-5}$
	Exp.	$1.13 \times 10^{-5} \pm 3.22 \times 10^{-6}$	$9.50 \times 10^{-6} \pm 1.58 \times 10^{-6}$	$4.85 \times 10^{-2} \pm 3.46 \times 10^{-2}$
GB	Sim.	$3.19 \times 10^{-5} \pm 1.15 \times 10^{-5}$	$3.21 \times 10^{-5} \pm 8.89 \times 10^{-6}$	$4.34 \times 10^{-2} \pm 1.97 \times 10^{-2}$
	Exp.	$3.71 \times 10^{-5} \pm 1.12 \times 10^{-5}$	$3.30 \times 10^{-5} \pm 6.64 \times 10^{-6}$	$3.16 \times 10^{-2} \pm 1.00 \times 10^{-2}$
LSTM	Sim.	$3.90 \times 10^{-5} \pm 1.23 \times 10^{-5}$	$3.90 \times 10^{-5} \pm 7.47 \times 10^{-6}$	$1.84 \times 10^{-2} \pm 7.32 \times 10^{-3}$
	Exp.	$3.56 \times 10^{-5} \pm 1.02 \times 10^{-5}$	$3.01 \times 10^{-5} \pm 9.47 \times 10^{-6}$	$2.16 \times 10^{-2} \pm 1.18 \times 10^{-2}$
RF	Sim.	$3.29 \times 10^{-5} \pm 1.27 \times 10^{-5}$	$3.49 \times 10^{-5} \pm 1.05 \times 10^{-5}$	$3.93 \times 10^{-2} \pm 2.08 \times 10^{-2}$
	Exp.	$4.67 \times 10^{-5} \pm 1.68 \times 10^{-5}$	$3.89 \times 10^{-5} \pm 7.06 \times 10^{-6}$	$2.85 \times 10^{-2} \pm 1.97 \times 10^{-2}$

Lower values are better.

The proposed algorithms were compared statistically as given in Table 4 by using only the test dataset. Tukey multiple comparison test was used to compare more than one group. As a result of this test, no significant statistical difference was found between only LSTM and RF algorithms. However, there was a significant statistical difference between the other groups. In this case, it shows that LSTM and RF algorithms approach the problem solution with the same perspective while other algorithms produce results independent of each other.

**Table 4:** Tukey HSD multiple comparison results for model MSE on the test dataset.

Group 1	Group 2	Mean Difference	p-value	Significant
CNN	GB	0.0043	0.9098	No
CNN	LSTM	-0.0015	0.9957	No
CNN	RF	0.0032	0.9628	No
GB	LSTM	-0.0058	0.8077	No
GB	RF	-0.0012	0.9978	No
LSTM	RF	0.0047	0.8921	No

The p-values indicate whether differences between model pairs are statistically significant ($\alpha = 0.05$).

## Discussions and conclusions

The precise determination of a mobile robot’s position is crucial for autonomous navigation. Odometry sensors provide position estimates based on wheel encoders, but their accuracy can degrade if wheels slip or rotate without forward motion. To mitigate these limitations, IMU sensors are commonly integrated, offering quaternion, angular, and linear velocities along three axes. When coupled with a robust estimation model, IMUs can deliver highly accurate position information, enhancing reliability while providing a cost-effective solution.

Predicting real-world robot positions based solely on noiseless simulation data is insufficient. Introducing noise to the input data significantly improved model estimation performance. In this study, 2,018 routes were generated to form the datasets for training, validation, and testing. Various architectures-including CNN, LSTM, RF, and GB-were employed for position estimation, with hyperparameters tuned using the validation data.

The superior performance of the CNN can be attributed to its ability to capture short-term temporal dependencies and complex multi-channel sensor correlations, as confirmed by the 1-step vs 3-step ablation study (Train loss: 0.0728 vs 0.0290, Test loss: 0.0739 vs 0.0282). This indicates that the CNN effectively exploits temporal information and multi-channel sensor correlations, which explains its superior accuracy compared to 1-step CNN and other models. In contrast, LSTM and RF models produced similar results but were less effective at exploiting these temporal features, explaining the observed performance gap. The slight decline in CNN performance on real-world data compared to simulation is likely due to unmodeled sensor noise, calibration errors, and environmental variations, highlighting the importance of domain adaptation and robust noise handling for practical deployment.

Compared to traditional methods such as Kalman and particle filters, which require careful noise modeling and may struggle with high-dimensional, multi-sensor inputs, CNNs and other deep learning models learn complex sensor relationships directly from data, enabling real-time inference with minimal manual tuning. While CNN achieved the best accuracy, all learned models offer advantages in adaptability, scalability, and reduced calibration effort, underscoring the potential benefits of data-driven approaches for mobile robot localization.

Several limitations should be noted. The study relies mainly on simulated data with added noise, and the real-world test set is relatively small, potentially limiting generalizability. Scaling the approach to dynamic environments, uneven terrain, or surfaces with variable friction may challenge model accuracy. Wheel slip, which is neglected in this study, could degrade performance in real deployments. Future work should incorporate larger real-world datasets, test in complex and dynamic environments, include wheel-slip compensation, and explore multi-sensor fusion techniques such as attention mechanisms. Model compression methods like pruning or quantization could further enhance computational efficiency.

Overall, the results indicate that IMU sensors, when combined with CNN-based estimation, can provide a reliable alternative for mobile robot positioning without requiring extensive additional sensing, offering promising directions for real-world applications.

## Data Availability

The datasets used and/or analysed during the current study available from the corresponding author on reasonable request.

## List of symbols

CNN: Convolutional neural network
LSTM: Long short-term memory network
GB: Gradient boosting
RF: Random forest
MAE: Mean absolute error
MSE: Mean squared error
RMSE: Root mean squared error
$R^2$: Coefficient of determination
m: Meter
rad: Radian
IMU: Inertial measurement unit
DDWMR: Differential drive wheeled mobile robot
ROS: Robot operating system
PPA: Pure pursuit algorithm

## References

[1] Boztas, G. & Aydogmus, O. Implementation of pure pursuit algorithm for nonholonomic mobile robot using robot operating system. *Balkan J. Electr. Comput. Eng.* **9**, 337–341. 10.17694/bajece.983350 (2021).

[2] Gareis, M. et al. Stocktaking robots, automatic inventory, and 3d product maps: The smart warehouse enabled by uhf-rfid synthetic aperture localization techniques. *IEEE Microwave Mag.* **22**, 57–68. 10.1109/MMM.2020.3042443 (2021).

[3] Gao, X. et al. Review of wheeled mobile robots’ navigation problems and application prospects in agriculture. *IEEE Access* **6**, 49248–49268. 10.1109/ACCESS.2018.2868848 (2018).

[4] Bärtschi, A. et al. Collaborative delivery with energy-constrained mobile robots. *Theoret. Comput. Sci.* **810**, 2–14. 10.1016/j.tcs.2017.04.018 (2020) (**Special issue on Structural Information and Communication Complexity.**).

[5] Namba, T. & Yamada, Y. Risks of deep reinforcement learning applied to fall prevention assist by autonomous mobile robots in the hospital. *Big Data Cogn. Comput.*10.3390/bdcc2020013 (2018).

[6] Fragapane, G., de Koster, R., Sgarbossa, F. & Strandhagen, J. O. Planning and control of autonomous mobile robots for intralogistics: Literature review and research agenda. *Eur. J. Oper. Res.* **294**, 405–426. 10.1016/j.ejor.2021.01.019 (2021).

[7] Fan, H., Hernandez Bennetts, V., Schaffernicht, E. & Lilienthal, A. J. Towards gas discrimination and mapping in emergency response scenarios using a mobile robot with an electronic nose. *Sensors*10.3390/s19030685 (2019). 30736489 10.3390/s19030685 PMC6387125

[8] Tuna, G., Gungor, V. C. & Gulez, K. An autonomous wireless sensor network deployment system using mobile robots for human existence detection in case of disasters. *Ad Hoc Netw.* **13**, 54–68. 10.1016/j.adhoc.2012.06.006 (2014).

[9] Schilling, K. & Jungius, C. Mobile robots for planetary exploration. *Control. Eng. Pract.* **4**, 513–524. 10.1016/0967-0661(96)00034-2 (1996).

[10] Jochum, E., Millar, P. & Nuñez, D. Sequence and chance: Design and control methods for entertainment robots. *Robot. Auton. Syst.* **87**, 372–380. 10.1016/j.robot.2016.08.019 (2017).

[11] Chen, Z., Liu, Y., He, W., Qiao, H. & Ji, H. Adaptive-neural-network-based trajectory tracking control for a nonholonomic wheeled mobile robot with velocity constraints. *IEEE Trans. Industr. Electron.* **68**, 5057–5067. 10.1109/TIE.2020.2989711 (2021).

[12] Greenberg, J. N. & Tan, X. Dynamic optical localization of a mobile robot using kalman filtering-based position prediction. *IEEE/ASME Trans. Mechatron.* **25**, 2483–2492. 10.1109/TMECH.2020.2980434 (2020).

[13] Su, Y., Wang, T., Shao, S., Yao, C. & Wang, Z. Gr-loam: Lidar-based sensor fusion slam for ground robots on complex terrain. *Robot. Auton. Syst.* **140**, 103759. 10.1016/j.robot.2021.103759 (2021).

[14] Bangaru, S. S., Wang, C., Busam, S. A. & Aghazadeh, F. Ann-based automated scaffold builder activity recognition through wearable emg and imu sensors. *Autom. Constr.* **126**, 103653. 10.1016/j.autcon.2021.103653 (2021).

[15] Ding, A., Zhang, Y., Zhu, L., Du, Y. & Ma, L. Recognition method research on rough handling of express parcels based on acceleration features and CNN. *Measurement*10.1016/j.measurement.2020.107942 (2020).

[16] Suri, K. & Gupta, R. Continuous sign language recognition from wearable IMUs using deep capsule networks and game theory. *Comput. Electr. Eng.* **78**, 493–503. 10.1016/j.compeleceng.2019.08.006 (2019).

[17] Peng, Y. et al. Dam behavior patterns in Japanese black beef cattle prior to calving: Automated detection using LSTM-RNN. *Comput. Electron. Agric.*10.1016/j.compag.2019.105178 (2020).

[18] Kim, W. Y., Seo, H. I. & Seo, D. H. Nine-Axis IMU-based Extended inertial odometry neural network. *Expert Syst. Appl.*10.1016/j.eswa.2021.115075 (2021).

[19] Zhao, J. & Obonyo, E. Convolutional long short-term memory model for recognizing construction workers’ postures from wearable inertial measurement units. *Adv. Eng. Inform.*10.1016/j.aei.2020.101177 (2020).

[20] Qaroush, A., Yassin, S., Al-Nubani, A. & Alqam, A. Smart, comfortable wearable system for recognizing Arabic Sign Language in real-time using IMUs and features-based fusion. *Expert Syst. Appl.* **184**, 115448. 10.1016/j.eswa.2021.115448 (2021).

[21] Kareem, Z. H., Bin Ramli, K. N., Malik, R. Q. & Zahra, M. M. A. Mobile phone user behavior’s recognition using gyroscope sensor and ML algorithms. *Mater. Today Proc.*10.1016/j.matpr.2021.04.639 (2021).

[22] Langroodi, A. K., Vahdatikhaki, F. & Doree, A. Activity recognition of construction equipment using fractional random forest. *Autom. Constr.*10.1016/j.autcon.2020.103465 (2021).

[23] Tan, T., Chiasson, D. P., Hu, H. & Shull, P. B. Influence of IMU position and orientation placement errors on ground reaction force estimation. *J. Biomech.*10.1016/j.jbiomech.2019.109416 (2019). 31630774 10.1016/j.jbiomech.2019.109416

[24] Liu, Q. et al. Classification of runners’ performance levels with concurrent prediction of biomechanical parameters using data from inertial measurement units. *J. Biomech.* **112**, 1–8. 10.1016/j.jbiomech.2020.110072 (2020). 10.1016/j.jbiomech.2020.110072 33075666

[25] Kiangala, S. K. & Wang, Z. An effective adaptive customization framework for small manufacturing plants using extreme gradient boosting-XGBoost and random forest ensemble learning algorithms in an Industry 4.0 environment. *Mach. Learn. Appl.* **4**, 100024. 10.1016/j.mlwa.2021.100024 (2021).

[26] Khnissi, K., Ben Jabeur, C. & Seddik, H. A smart mobile robot commands predictor using recursive neural network. *Robot. Auton. Syst.* **131**, 103593. 10.1016/j.robot.2020.103593 (2020).

[27] Szegedy, C. *et al.* Going deeper with convolutions. In *2015 IEEE Conference on Computer Vision and Pattern Recognition (CVPR)*, 1–9, 10.1109/CVPR.2015.7298594 (2015).

[28] He, K., Zhang, X., Ren, S. & Sun, J. Delving deep into rectifiers: Surpassing human-level performance on imagenet classification (2015). arXiv:1502.01852.

[29] Hochreiter, S. & Schmidhuber, J. Long short-term memory. *Neural Comput.* **9**, 1735–1780. 10.1162/neco.1997.9.8.1735 (1997). 9377276 10.1162/neco.1997.9.8.1735

[30] Bingol, M. C. & Aydogmus, O. Practical application of a safe human-robot interaction software. *Ind. Robot.* **47**, 359–368. 10.1108/IR-09-2019-0180 (2020).

[31] Bingol, M. C. & Aydogmus, O. Performing predefined tasks using the human-robot interaction on speech recognition for an industrial robot. *Eng. Appl. Artif. Intell.* **95**, 103903. 10.1016/j.engappai.2020.103903 (2020).

[32] Géron, A. *Hands-on Machine Learning with Scikit-Learn, Keras, and TensorFlow: Concepts, Tools, and Techniques to Build Intelligent Systems* (O’Reilly Media, 2019).

